# Nanosilica treatment enables moisture-resistant hydrophobic arc welding covered electrodes

**DOI:** 10.1038/s41598-023-37164-3

**Published:** 2023-06-19

**Authors:** Mohammadreza Pasandeh, Majid Pouranvari

**Affiliations:** 1 Department of Materials and Textile Engineering, Faculty of Engineering, Razi University, Kermanshah, Iran; 2 Department of Materials Science and Engineering, Sharif University of Technology, Tehran, 11365-9466 Iran

**Keywords:** Synthesis and processing, Metals and alloys

## Abstract

Controlling the moisture content of the electrode-covering is crucial in the production of defect-free, high-quality welds during shielded metal arc welding of steels. The welding industry has long faced the challenge of the high susceptibility of basic electrodes (e.g., E7018) to moisture absorption. In this paper, we demonstrate that applying a nanosilica coating to the surface of the E7018 electrode-covering using a dip-coating technique can effectively reduce the moisture absorption capability of the electrode-covering. The moisture measurement results before and after exposure to a moist environment of 80% humidity and a temperature of 27 °C for 9 h indicate that the moisture absorption values of conventional and nano-treated E7018 electrodes during exposure are 0.67 wt% and 0.03 wt%, respectively. While reducing the size of the pores on the surface of the electrode-covering can to some extent enhance the resistance to moisture absorption, it has been identified that turning the wetting behavior of the electrode-covering surface from hydrophilic to hydrophobic by the nanosilica coating is the most effective mechanism that contributes to the enhanced moisture absorption resistance of the nanosilica-treated electrode-covering. The results indicate that this approach does not have any deleterious effects on the chemical analysis and tensile properties of the weld metal. This simple modification to the electrode-covering can be generally applied to a wide range of electrode-covering types to produce hydrophobic, moisture-resistant electrodes.

## Introduction

Shielded metal arc welding (SMAW) is a highly versatile manufacturing technology that plays a vital role in various industrial applications, including the construction of buildings, bridges, pipelines, pressure vessels, ships, offshore structures, and submerged marine structures^1–6^. While SMAW can be used for welding non-ferrous materials, it is particularly well-suited for welding ferrous materials, such as cast iron, steel, and stainless steel. Its ability to produce high-quality welds in challenging conditions has made it a popular choice in a wide range of industries. However, as it is well documented in the literature^7,8^, the presence of hydrogen in the fusion zone during welding of steels can be dangerous as it causes cold cracking phenomena in both heat affected zone and the fusion zone, which are responsible for losses of life and property due to catastrophic failure of welded steel structure. Hydrogen-induced cold cracking is a significant weldability issue associated with high-strength steels^9–11^. Therefore, the increasing demand for high-strength steels has led to a greater need for low-hydrogen welding technologies to mitigate the risk of cold cracking.

It has been identified that the primary source of hydrogen in the weld metal is the decomposition products of the electrode covering in SMAW. The decomposition of CaCO_3_-containing basic electrode covering produces a gaseous shield low in H_2_^8^. Therefore, the use of basic electrode covering is the key approach to reducing the risk of cold cracking during welding of high-strength steels. Although basic electrode covering is a low-hydrogen welding consumable, it is susceptible to moisture pickup when exposed to the atmosphere^12^. It has been identified that the primary source of hydrogen in SMAW is the moisture of the electrode covering. In addition to its detrimental effect on weld cracking, moisture pickup can degrade weld quality by promoting the formation of subsurface porosity, which requires X-ray inspection or destructive testing. Moreover, high moisture can lead to a rough weld surface^13^. Therefore, controlling the moisture content of the basic electrode covering is key to obtaining high-quality arc welds. It requires careful handling and storage methods to prevent moisture absorption, as well as baking the electrodes at a temperature in the range of 340–400 °C^14^. As a result, these electrodes can only be exposed to ambient conditions for a limited time before the flux absorbs moisture from the air and has to be baked again to reduce the moisture content. However, proper storage and baking treatment are costly solutions. Therefore, covered electrodes with high resistance to moisture reabsorption are designed to control the hydrogen content of the weld metal.

According to AWS A5.1^15^, the so-called moisture-resistant basic coated electrodes, which are designated by adding the suffix letter “R” after the four-digit classification number, should have a moisture content after not less than 9 h of exposure to an environment of 27 °C and 80% relative humidity that is not higher than 0.4 wt%. The moisture-resistant basic electrodes are developed based on the optimized granulometry of the coating flux ingredient, newly developed binder system, and correct procedure used for removing almost the entire quantity of moisture from the flux^16^. Modifying the flux-binder system not only can affect the physicochemical and thermophysical behavior of the slag^17,18^, but it can also modify the resistance to moisture absorption of the coating. For example, Barringer and Eagar^19^ patented a welding flux-binder system based on a hydrolyzed and polymerized organometallic compound using a sol–gel method. Although they did not demonstrate the moisture absorption capability of the electrode coating, they reported that the resulting welding flux binder and flux are non-hygroscopic. However, the flux-binder system disclosed by Barringer and Eagar^19^ requires high temperatures (e.g., 750–800 °C) to set the binder.

Crockett^20^ and Dallam and Karogal^21^ showed that incorporating colloidal nano SiO_2_ in the binder system can reduce the hygroscopic moisture pickup of the electrode covering. The moisture absorption capability can be very low (0.02–0.04 wt% after 24 h of exposure to a moist environment of 80% humidity and a temperature of 27 °C) in covered electrodes with nanosilica-containing binders. Fattahi et al.^22^ showed that increasing the colloidal nanosilica content of the binder system from 0 to 30 wt% reduces the weld metal diffusible hydrogen from about 8 mL/100g to below 4 mL/100g. However, the binder/flux system requires a long high-temperature drying process. Vaz et al.^23^ produced a new binder system in which the usual binders (potassium and sodium silicates) were replaced by polymers. They showed that this approach can produce a low-hydrogen (i.e., less than 4 mL/100 g) impermeable covered electrode. It is noteworthy that generally, binder-based modifications to electrode covering are costly approaches. Tomków et al.^24^ demonstrated that applying a paraffin wax waterproof coating to the electrode surface is successful in reducing the diffusible hydrogen content in the weld metal during underwater welding by 35%.

With the increased utilization of advanced high-strength steels in engineering applications, there is a need to develop low-cost alternatives for producing moisture-resistant electrodes. One of the main mechanisms of moisture pickup during exposure is the physical absorption of water molecules through the pores of the coating surface. Therefore, producing an absorption barrier layer on the surface of the coating can be an effective approach to control moisture pickup during environmental exposure. Additionally, the presence of hygroscopic species (such as lime) in the electrode covering can enhance moisture absorption. Thus, producing a hydrophobic absorption-barrier layer on the surface of the coating can be a practical approach to controlling moisture pickup during environmental exposure.

In this work, we showcase the effectiveness of this approach by applying a nano-structured hydrophobic coat on the surface of a welding electrode with a basic covering. We chose a nanosilica coating due to its specific characteristics: (i) SiO_2_ nanoparticles can exhibit hydrophobic characteristics^25^, (ii) SiO_2_ is insoluble in water, and (iii) SiO_2_ is not expensive. To enhance resistance to moisture absorption, we applied a nanosilica coating on the surface of the E7018 electrode-covering using a simple dip-coating method. We investigated the effect of this nanotechnology-treated approach on the moisture absorption capability of the electrode-covering.

## Experimental procedure

### Fabrication of nano-treated electrode coating

The E7018 basic welding electrode, one of the most commonly used SMAW electrodes, was used as the base electrode and received in a carton box. To ensure consistency in moisture levels, the electrodes were first dried at 350 °C for 2 h. In this research, a dip-coating technique was used to produce a thin SiO_2_ film on the surface of the E7018 electrode-covering. Therefore, a stable aqueous colloidal nanosilica sol (i.e., a suspension of silicon dioxide nanoparticles in water) was prepared. To produce a stable colloidal nanosilica solution, the size of the nanoparticles, which affects the gravity of the particles, and the solution pH, which determines the surface charge of the particles, should be controlled. The initial experiments showed that using nanoparticles coarser than 40 nm led to sedimentation of the nanoparticles in the nano-solution. Therefore, the suspension was prepared by directly adding the required amount of 20–40 nm SiO_2_ nanoparticles, which comprised 25–30 wt% into distilled water. It has been shown that increasing the pH can change the nature of the forces between nanoparticles from attractive-dominant to repulsive-dominant, resulting in a stable colloidal solution without agglomeration of nanoparticles^26^. The pH of the solution was adjusted by adding Na_2_O to the solution via ${\text{Na}}_{2} {\text{O}} + {\text{ H}}_{2} {\text{O}} \to {\text{NaOH}}$ reaction. The amount of Na_2_O was chosen to produce a solution with a pH in the range of 10–11. This pH range ensured the formation of a stable, clear colloidal nanosilica solution. The composition of the produced stable colloidal nanosilica solution is shown in Table 1.

**Table 1:** Chemical composition of Nanosilica solution (wt%).

SiO_2_	Na_2_O	Distilled water
25 – 30	0.3–0.6	Base

To produce a nano-treated electrode covering, the dip-coating process was utilized. This involved immersing a pre-dried E7018 electrode into a stable colloidal nanosilica solution for a period of 10 seconds, after which the nano-treated electrode was dried at 200 °C for 1 h. The dipping time was sufficient to produce a uniform thin film on the electrode surface. The baking time and temperature were chosen to ensure complete evaporation of the solvent, allowing the coating to solidify and adhere to the object effectively.

### Characterization of nano-treated electrode coating

The moisture content of the conventional and nano-treated electrode was a critical parameter that was measured in this study to evaluate the effectiveness of the nanosilica coating in reducing moisture absorption. The moisture analysis was performed based on a weight-loss basis, following the guidelines of the AWS 5.1 standard, after exposing the electrode-covering to an environment of 80% humidity for a period of 9 h. This testing procedure was repeated three times for each type of electrode to ensure the accuracy and reliability of the results.

To gain a better understanding of the effect of the nanosilica coating on the electrode surface, the surface morphology of both treated and non-treated electrodes was examined using field-emission scanning electron microscopy (FESEM). Additionally, the water wettability of the conventional and nano-treated electrode-covering surfaces was measured using the sessile drop technique. Water wettability is an essential parameter for evaluating moisture absorption resistance, as it reflects the ability of the electrode surface to repel water droplets. For this test, a 3 µl water droplet was placed on the electrode surface, and an image was taken at the moment the drop became steady. The water droplet shape and the left and right water contact angles were determined with an optical contact angle measuring and contour analysis system of the OCA 100 (DataPhysics Instruments, Filderstadt, Germany) at room temperature. The contact angle is the angle formed at the intersection of the water droplet and the electrode surface. Specifically, the right and left contact angles are the tangent angles at the intersections of the water droplet outline and the baseline, which is the contact point between the water droplet and the substrate. These angles provide a quantitative measure of the wetting properties of the electrode surface, with higher contact angles indicating greater water repellency. The contact angle was measured at least 15 times for each electrode type using an automatic method of OCA 100, providing a reliable and accurate measurement of the wetting angle.

To evaluate the effect of the nanosilica coating on the composition and properties of the weld metal, shielded metal arc welding (SMAW) experiments were conducted using both conventional and nano-treated E7018 electrodes on 15 mm thick ST37 carbon steel plates. The plates were first cut and machined to create a double V groove butt joint configuration with a groove angle of 60 degrees. Three passes were used to fill each side of the joint, and the joint was also back welded. The welding experiments were performed by a skilled welder to assess the ease of welding using the nano-treated electrode, and it was found that the arc initiation and stability were not affected by the presence of the nanosilica coating. The chemical composition of the weld metal was determined using spark emission spectrometry. To investigate the effect of the nanosilica coating on joint properties, transverse tensile test samples and longitudinal all weld metal samples were machined from the weld coupon according to the AWS B4.0/B4.0M:2000 standard^27^. In the case of the transverse tensile test, the tensile strength and location of the failure were determined, while in the case of the all weld metal tensile test, the yield strength, tensile strength, and total elongation of the weld metal were measured.

## Results and discussion

### Moisture absorption capability of the electrodes

The moisture content of the conventional and nano-treated electrodes was measured before and after exposure to a moist environment of 80% humidity and a temperature of 27 °C for 9 h.

**Figure 1:**
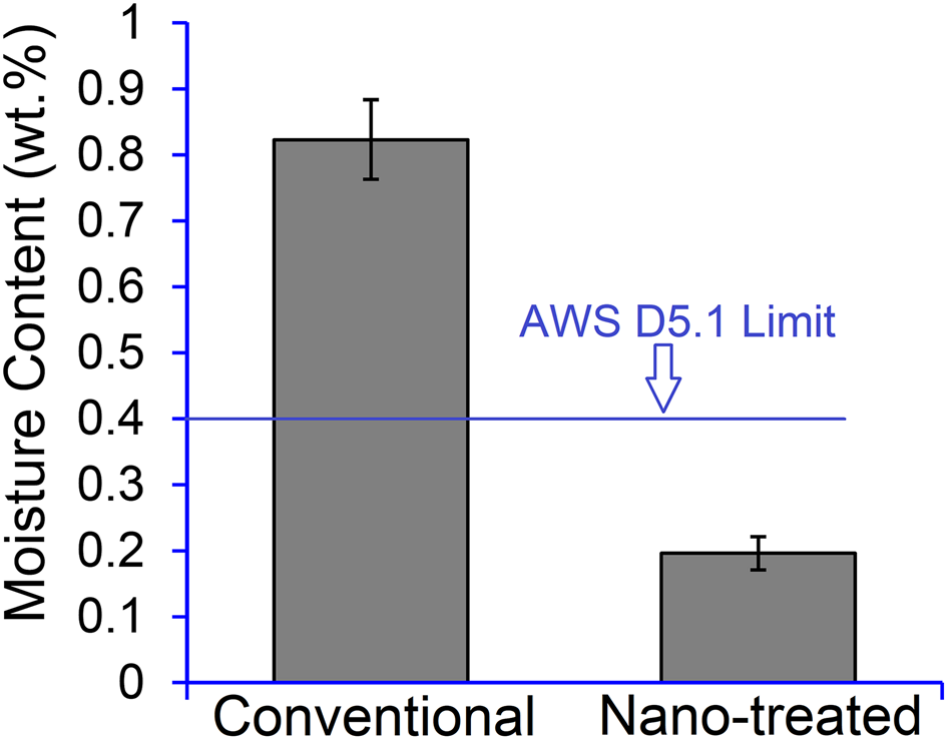
Moisture content of the E7018 electrode-covering after exposure: conventional versus nano-treated silica-coated electrode-covering. The maximum allowable moisture content of the moisture-resistant E7018M electrode based on the AWS D5.1 is also shown. The error bars represent the standard deviation of the data.

1. *Conventional electrode:* The initial moisture content of the conventional electrodes (i.e., before exposure to a moist environment), measured after drying at 350 °C for 2 h, was about 0.15 wt%. The as-exposed moisture content of the conventional non-treated electrodes was then measured after exposure to the moist environment. The average as-exposed moisture content of the conventional E7018 welding electrodes was 0.82 wt%. The moisture measurement results before and after exposure indicate that the moisture absorption of conventional E7018 electrodes during exposure was 0.67 wt%.
2. *Nano-treated electrodes:* The initial moisture content of the electrodes was measured just after the final step of the coating process, which involved drying at 200 °C for 1 h. The average moisture content was 0.16 wt%. The as-exposed moisture content of the nano-treated electrodes was also measured just after the final step of the coating process. The electrode-covering was then exposed to the moist environment for 9 h. The as-exposed moisture content of the nano-treated E7018 welding electrodes was 0.19 wt%. Figure 1 compares the average as-exposed moisture content of the conventional and nano-treated E7018 welding electrodes after the moisture absorption test. The as-exposed moisture in the nano-treated electrode decreased by 76% compared to the conventional non-treated electrode. The average measured moisture content of the nano-treated E7018, which was 0.19 wt%, is much lower than the maximum allowable moisture content of the moisture-resistant E7018M electrodes, which is 0.4 wt% as specified in AWS A5.1. The moisture measurement results before and after exposure indicate that the moisture absorption of nano-treated E7018 electrodes during exposure was 0.03 wt%. This confirms the effectiveness of the nano-SiO_2_ coating in reducing moisture absorption. This approach can change the current recommendations for baking electrodes before welding practice.

### The mechanisms for enhanced moisture absorption resistance of the nano-treated coated electrode

**Figure 2:**
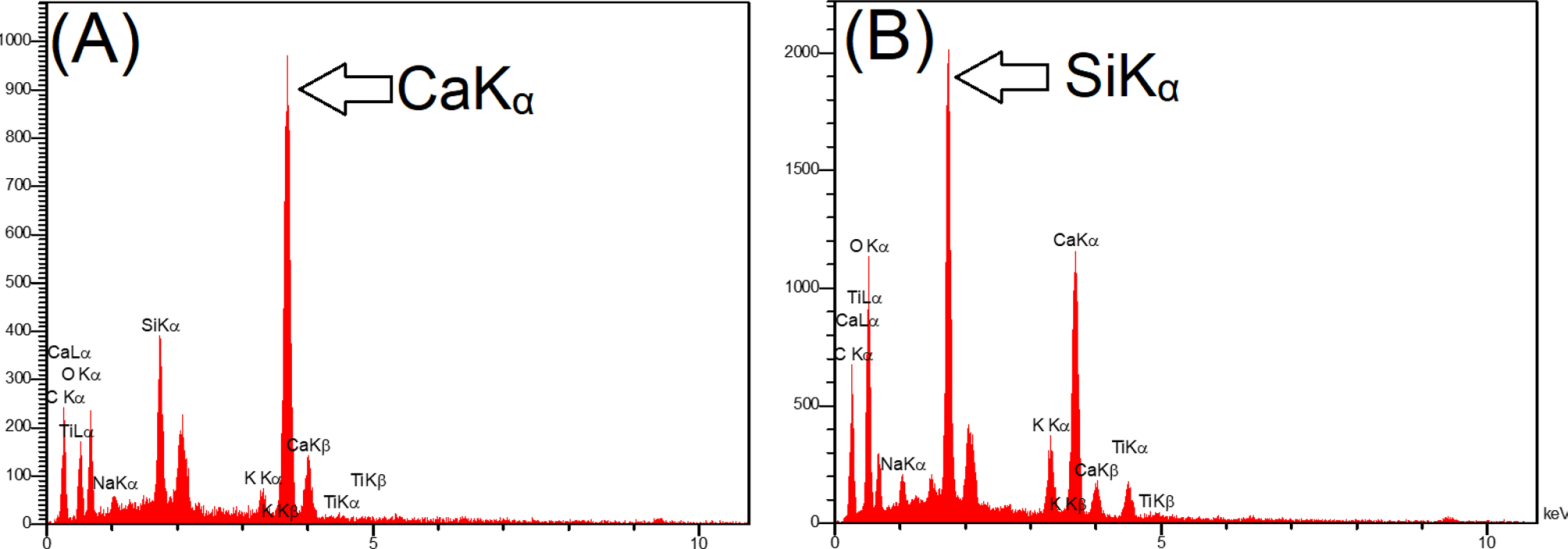
EDS-FESEM spectra for covered electrode surface in (**A**) conventional non-treated electrode-covering and (**B**) nano-treated silica-coated electrode-covering.

**Figure 3:**
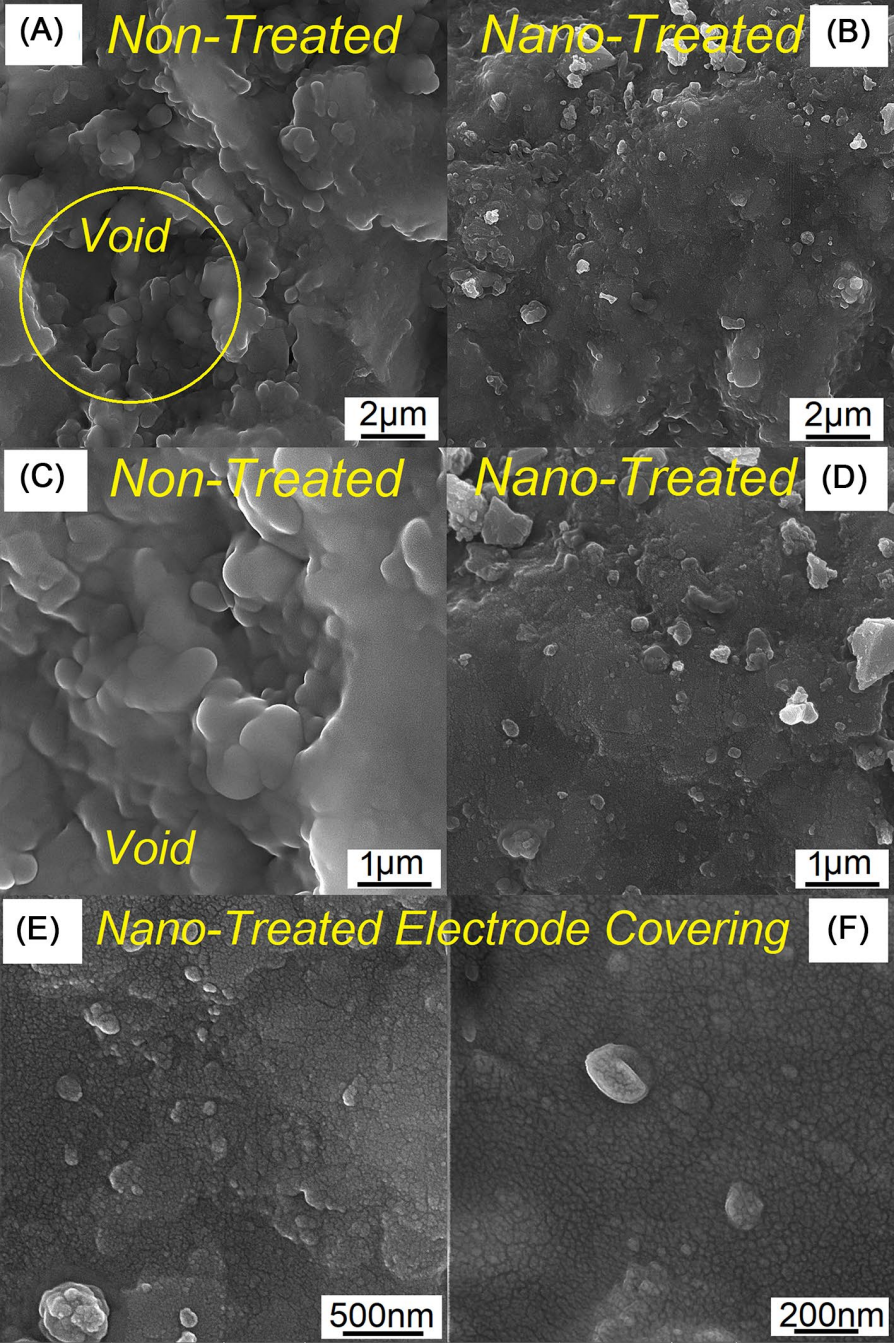
Morphology of the electrode surface: (**A** and **C**) conventional non-treated electrode, (**B**, **D**–**F**) nano-treated silica coated electrode.

To investigate the reasons behind the improved moisture absorption resistance of the nanosilica coated electrode-covering, the surface characteristics of both non-treated and nano-treated electrodes were examined. Figure 2A–B shows the FESEM-EDS spectra of the conventional and nano-treated electrode surfaces, respectively. The peak of Ca and Si in the conventional E7018 electrode is due to the presence of a high amount of CaCO_3_, CaF_2_, and SiO_2_ in its basic electrode-covering composition. However, the FESEM-EDS spectrum of the nano-treated electrode exhibited a very high-intensity peak of Si, confirming the formation of a thin nanosilica coat on the electrode-covering. Figure 3 shows the surface morphology of the conventional E7018 electrode and the nano-treated silica coated E7018 electrode. According to Fig. 3A–D, the surface voids of the nano-treated electrode are smaller than those of the conventional electrode. According to Fig. 3E–F, the presence of a nearly closed-packed array of nano-sized particles on the surface of the nano-treated electrode-covering is evident. The size of nanoparticles is in the range of 20–40 nm, corresponding to the size of nanoparticles in colloidal nanosilica solution. The presence of nano-sized particles can fill the surface porosity/voids of the electrode-covering and hence enhance the anti-absorption characteristics of the nano-treated electrode-covering.

According to the Lucas-Washburn theory^28^ for fluid absorption on porous media, the total liquid volume absorbed per unit area is related to the number and size of the pores. Therefore, increasing the size and number of the pores increases fluid absorption. Consequently, it is believed that applying the nanosilica coating on the surface of the electrode-covering, which reduces the size of the pores/voids, can decelerate water absorption through the pores. However, this mechanism cannot contribute largely to the observed reduction in moisture absorption. Indeed, the most crucial role of the nanosilica coating is its effect on the wetting behavior of the surface.

Generally, decreasing the surface wettability can discourage water absorption^29^. Therefore, an effective approach to enhance resistance to moisture absorption is to produce a hydrophobic surface. In this study, we compared the wetting behavior of both conventional and nano-treated electrodes.

**Figure 4:**
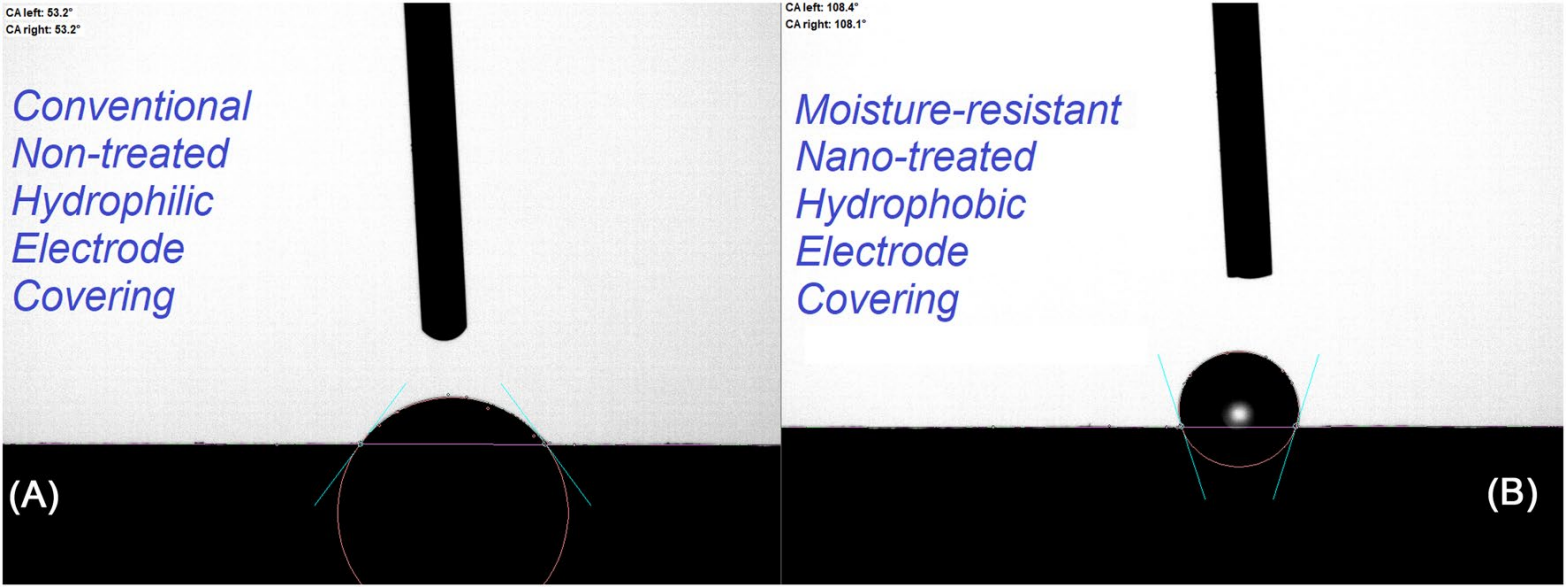
Water droplet profile on the surface of the (**A**) conventional non-treated electrode-covering and (**B**) nano-treated silica-coated electrode-covering.

**Figure 5:**
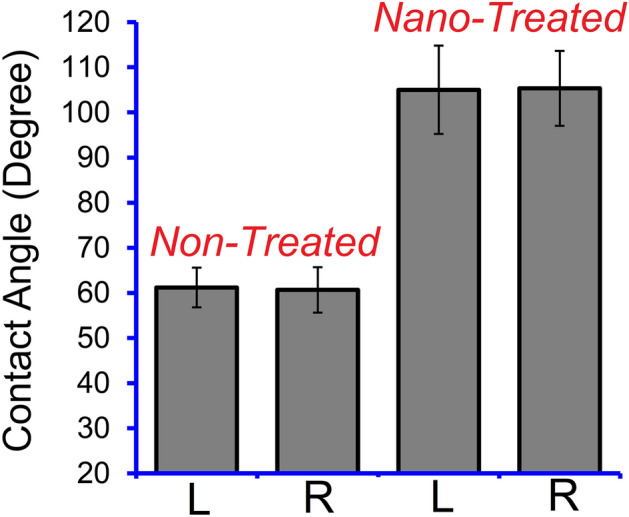
Left (L) and right (R) water contact angle on the surface of the conventional non-treated and nano-treated silica-coated electrode-covering. The error bars represent the standard deviation of the data.

Figure 4 compares the water droplet profiles on the surface of the conventional and nano-treated electrodes, demonstrating the hydrophobic characteristics of the nanosilica coated E7018 electrode. Figure 5 shows the results of several contact angle measurements. As shown, the average contact angle for the non-treated conventional electrode is 61°, indicating a hydrophilic behavior. However, the average contact angle for the nano-treated SiO_2_ coated electrode is 105°, indicating a hydrophobic behavior. The hydrophobic nature of the surface of the nano-treated electrode can be attributed to the hydrophobic nature of the SiO_2_ nanoparticles. The transformation of the wetting behavior of the electrode-covering surface from hydrophilic to hydrophobic using a thin film of SiO_2_ nanoparticles can also contribute to the lower moisture absorption capability of the nano-treated electrode-covering.

### Effect of nanosilica coating on the weld properties

It is worth noting that the presence of a nanosilica thin film on the surface of the electrode covering does not affect the composition of the weld metal. Table 2 shows the chemical composition of the weld metal produced using both conventional and nano-treated silica-coated E7018 electrodes, indicating that the nanosilica coating on the electrode covering did not impact the chemical composition of the weld metal. Therefore, it is expected that the nanosilica coating will not have a detrimental effect on the microstructure and mechanical properties of the weld metal. Moreover, transverse tensile testing of the welded samples using nano-treated electrodes showed that the failure location of the welded samples was in the base metal and the average joint strength was 395 MPa, which is equivalent to the tensile strength of the ST37 base metal. This proves that the presence of a nanosilica coating on the surface of the electrode covering does not have any detrimental effect on joint properties.

**Table 2:** Chemical composition (wt%) of the weld metal produced using both conventional non-treated and nano-treated E7018.

Elements	C	Mn	Si	Ni	Cr	Mo	S	P
Non-treated	0.09	1.10	0.33	0.04	0.07	0.02	0.01	0.01
Nano-treated	0.09	1.14	0.29	0.05	0.07	0.02	0.01	0.01

To further explore the effect of the nanosilica coating on weld performance, we investigated the tensile properties of the weld metal. Table 3 shows the tensile properties of the weld metal produced using both conventional non-treated and nano-treated E7018 electrodes. According to Table 3, the tensile properties of the conventional E7018 and the nano-treated E7018 in terms of yield strength, tensile strength, and elongation are the same and meet the minimum requirements of the tensile test for E7018 and E7018M.

**Table 3:** Tensile properties of the weld metal produced using both conventional non-treated and nano-treated E7018.

Tensile properties	Yield strength (MPa)	Tensile strength (MPa)	Elongation (%)
Non-treated	450	553	35
Nano-treated	465	548	35

### Final remark

The cost-effectiveness of the nanosilica coating approach for improving moisture absorption resistance depends on the cost of coating materials, coating process, and potential savings from reduced preheating and baking before welding. The coating materials are inexpensive as they use abundant nano-sized silica particles. The dip-coating process is a simple, easy and cost-effective. Also, in contrast to the approaches based on the modification of flux/binder system which generally requires a long and high temperature drying process, the nanosilica coating of the onventional SMAW electrode covering short requires a short and low-temperature drying process after coating to allow silica particles to adhere to the electrode covering. The reduction in preheating and baking needs can provide significant cost savings, especially for large-scale welding operations. Therefore, nanosilica coating approach is a cost-effective solution that can reduce overall costs and improve productivity.

## Conclusions

Controlling moisture absorption in the basic electrode-covering is crucial for producing high-quality, porosity-free welds with improved resistance to hydrogen cracking. In this work, a cost-effective and straightforward nanotechnology-based approach was used to enhance the moisture resistance of the E7018 electrode-covering. By applying a thin film of nanosilica coating on the surface of the electrode-covering, the moisture absorption of the electrode was significantly reduced compared to that of the conventional E7018 electrode. While the nanosilica coating can reduce the volume fraction of existing pores on the surface of the electrode-covering, the conversion of the surface of the electrode covering from hydrophilic to hydrophobic nature is the most critical factor contributing to the moisture-resistant characteristics of the electrode-covering. The results showed that the use of nanosilica coating on the surface of the electrode-covering did not have a deleterious effect on the chemical analysis and tensile properties of the weld metal. This work highlights the potential of nanotechnology-based treatments to improve the performance of welding consumables. The use of nanosilica coating on welding electrodes could potentially reduce the need for baking before welding, which can save time and energy while improving the ease and convenience of welding operations. These findings could have significant implications for the manufacturing and construction industries, where high-quality, reliable welds are crucial.

## Data Availability

The datasets used and/or analysed during the current study are available from the corresponding author on reasonable request.
